# Supplementary material for: Polyelectrolyte complex micelles embedded in hyaluronic acid gels enable local, targeted miR-92a inhibition to accelerate diabetic wound repair
Source: bioRxiv. 2025 Nov 28:2025.11.25.690510. Preprint. [Version 1] doi: 10.1101/2025.11.25.690510 (PMC12704000; doi:10.1101/2025.11.25.690510)
Supplement: Supplement 1 [file media-1.pdf]

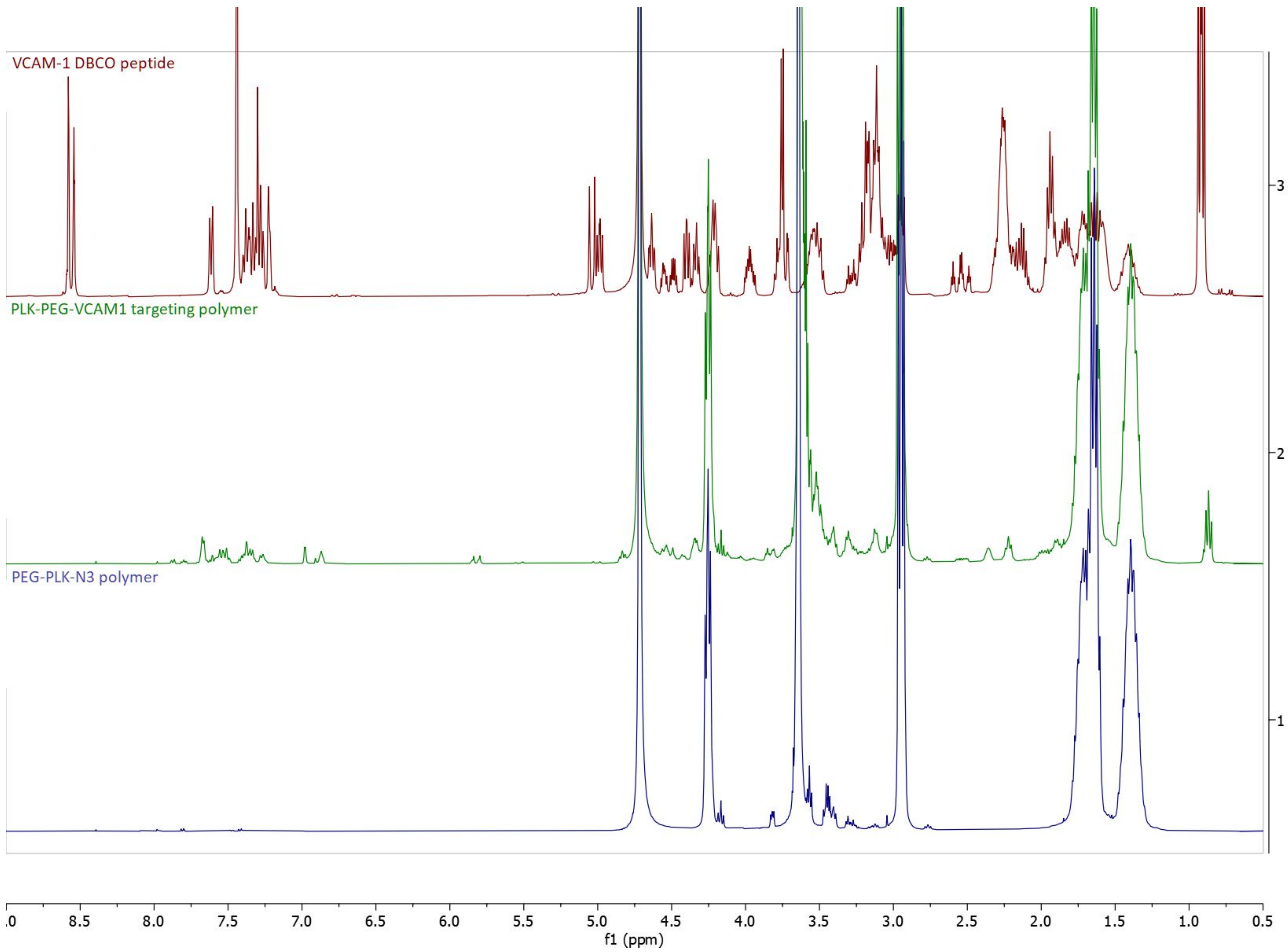

Supplementary Figure S1: NMR Showing conjugation of PEG2000-poly-L-lysine(30) with VCAM-1 targeting peptide, post purification

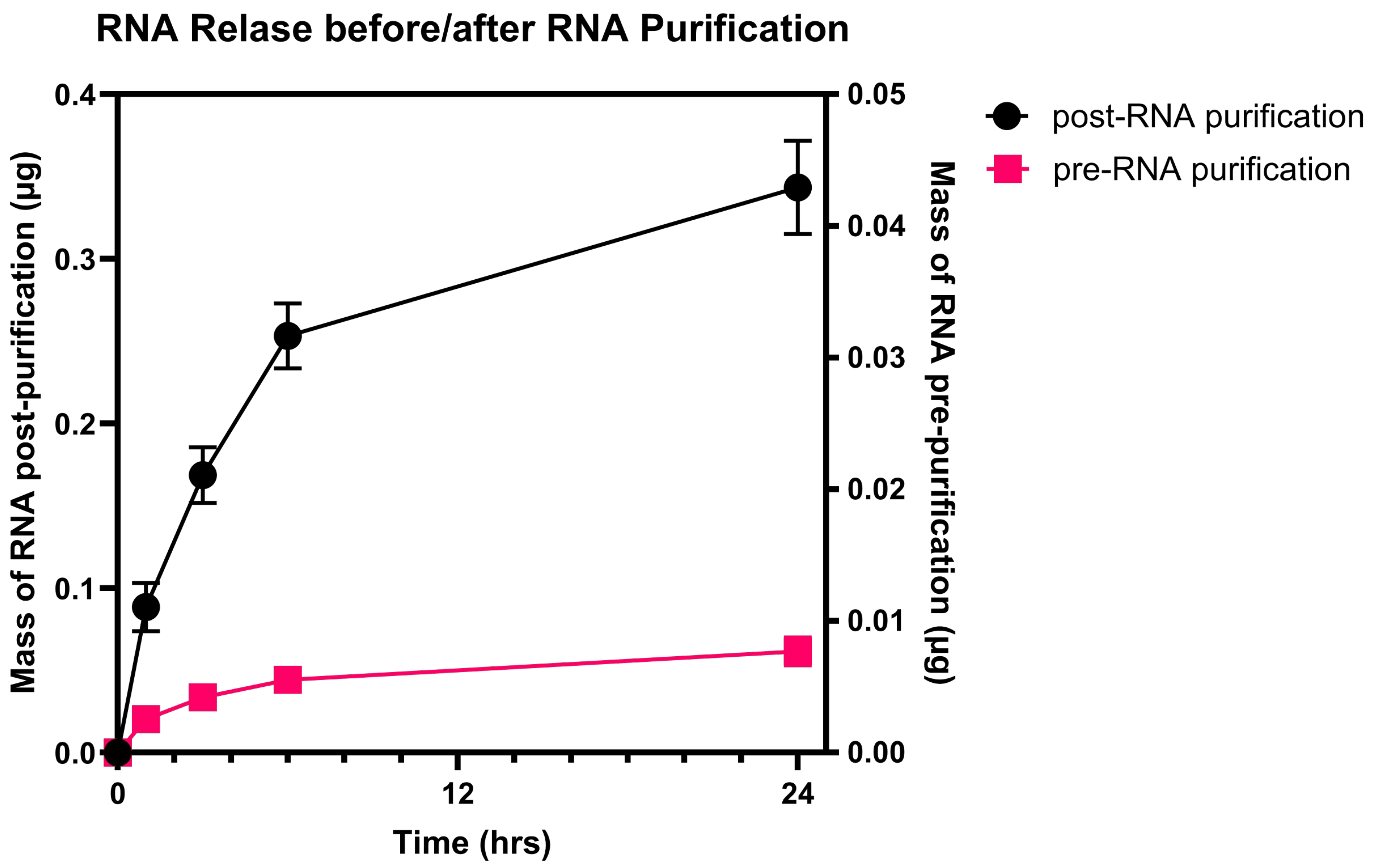

Supplementary Figure S2: RNA release curves from 150µL of gel pre- and post-RNA purification, as measured by RiboGreen Assay. Only ~2% of released RNA is detectable prior to purification, implying an encapsulation efficiency of 98% for miRNA released from PCM-gels.

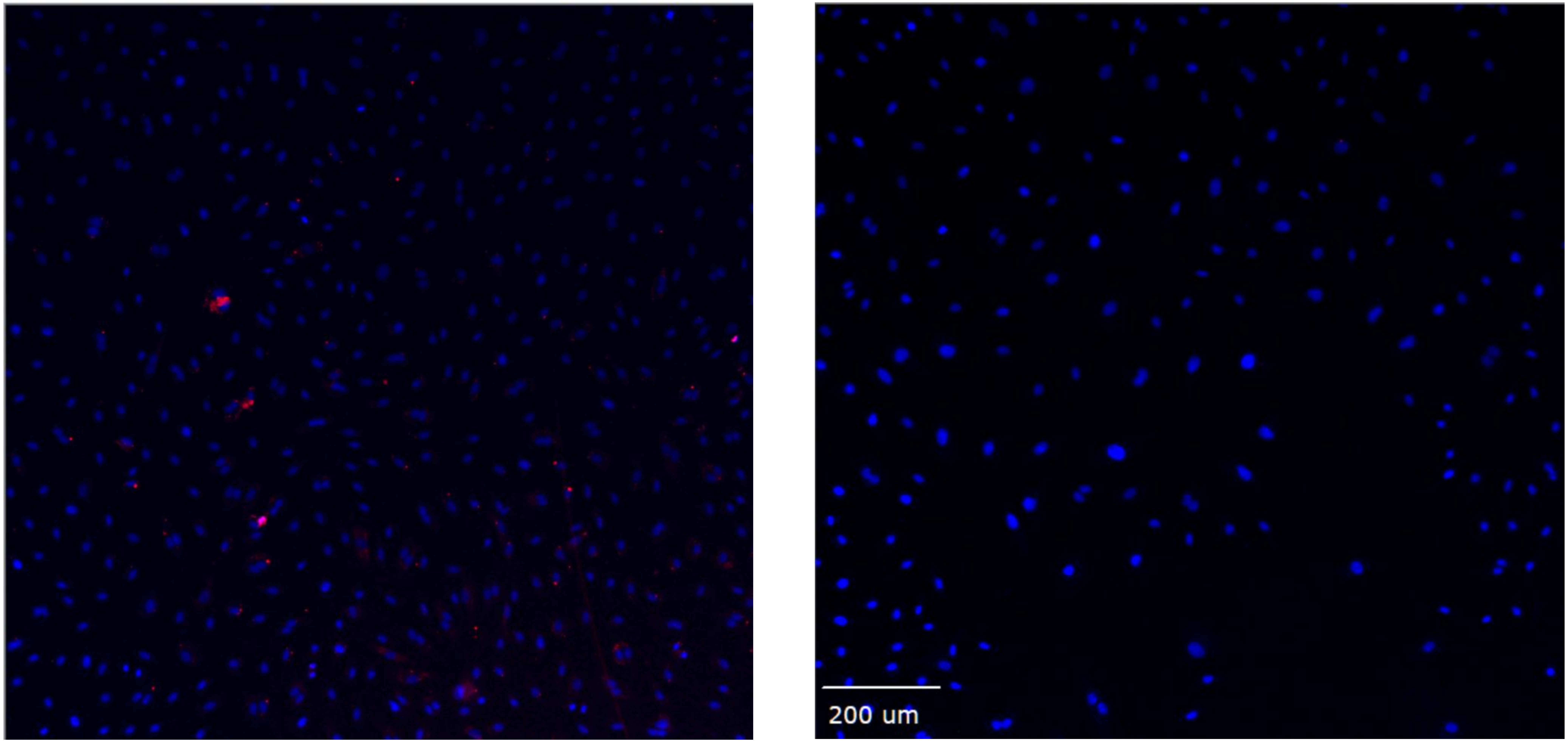

Supplementary Figure S3: Cy3-tagged miRNA inhibitor uptake in HAECs. Cells were treated for 3 hours with PCM-gels containing Cy3-labeled RNA (left) or no gels (right), and left to incubate overnight. Red = Cy3, Blue = DAPI

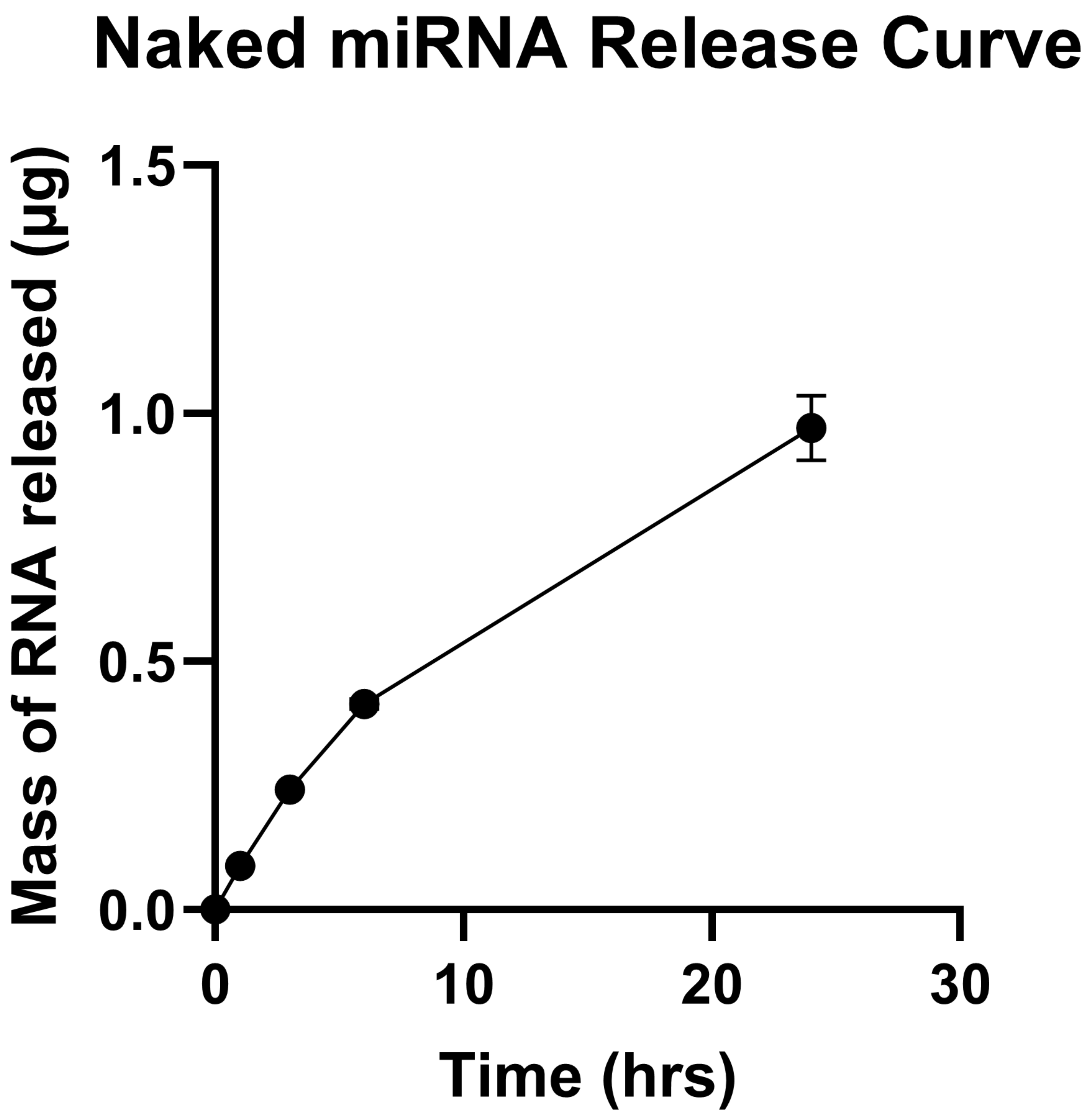

Supplementary Figure S4: Release curve for naked miRNA from gel formulation. No RNA purification was performed prior to measurement; diffused miRNA inhibitor was directly quantified by RiboGreen assay.

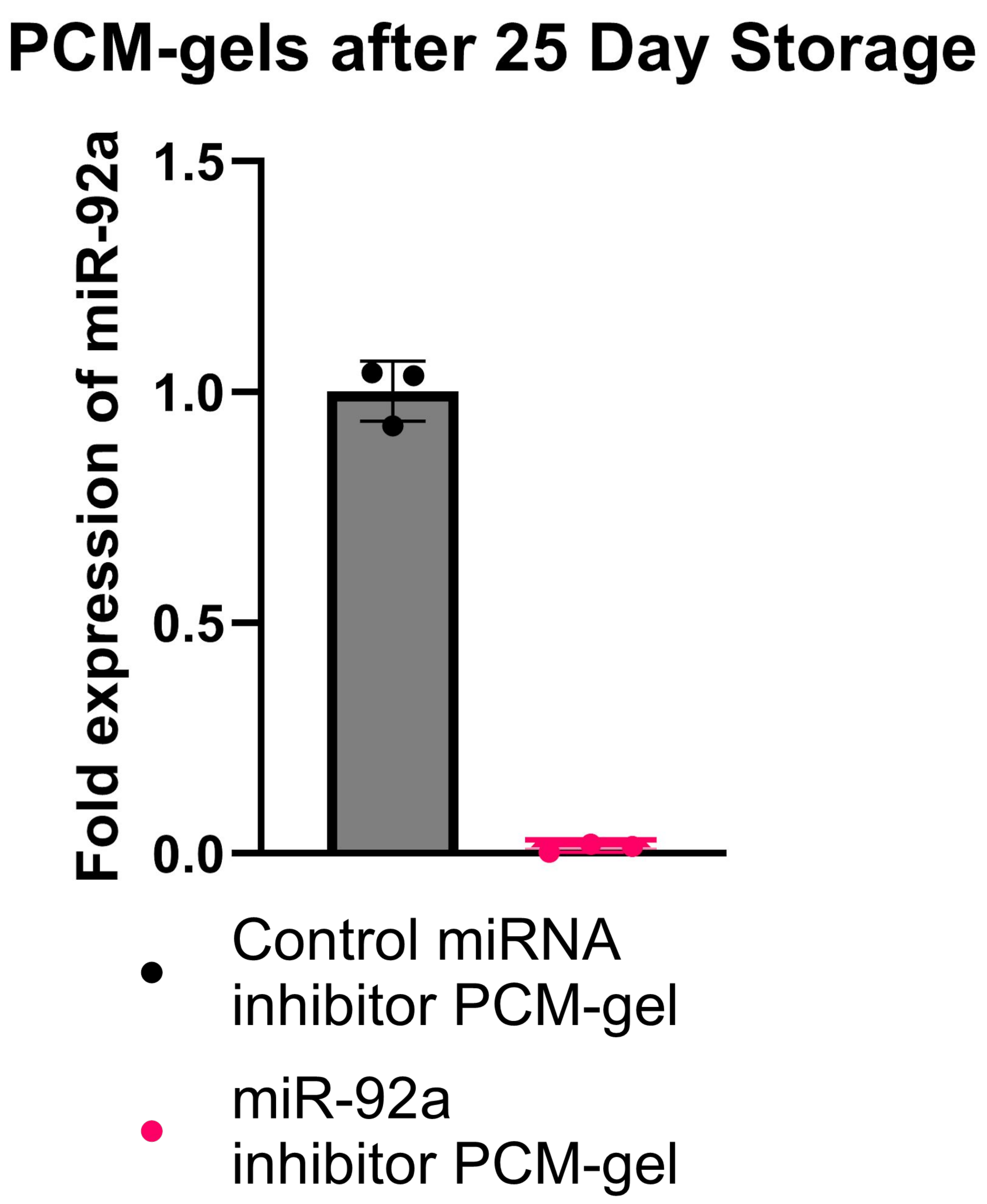

Supplementary Figure S5: miR-92a silencing efficacy of PCM-gels stored for 25 days at 4°C. HAECs were pre-treated with LPS for 3 hours, then treated with PCM-gels overnight before RNA extraction.

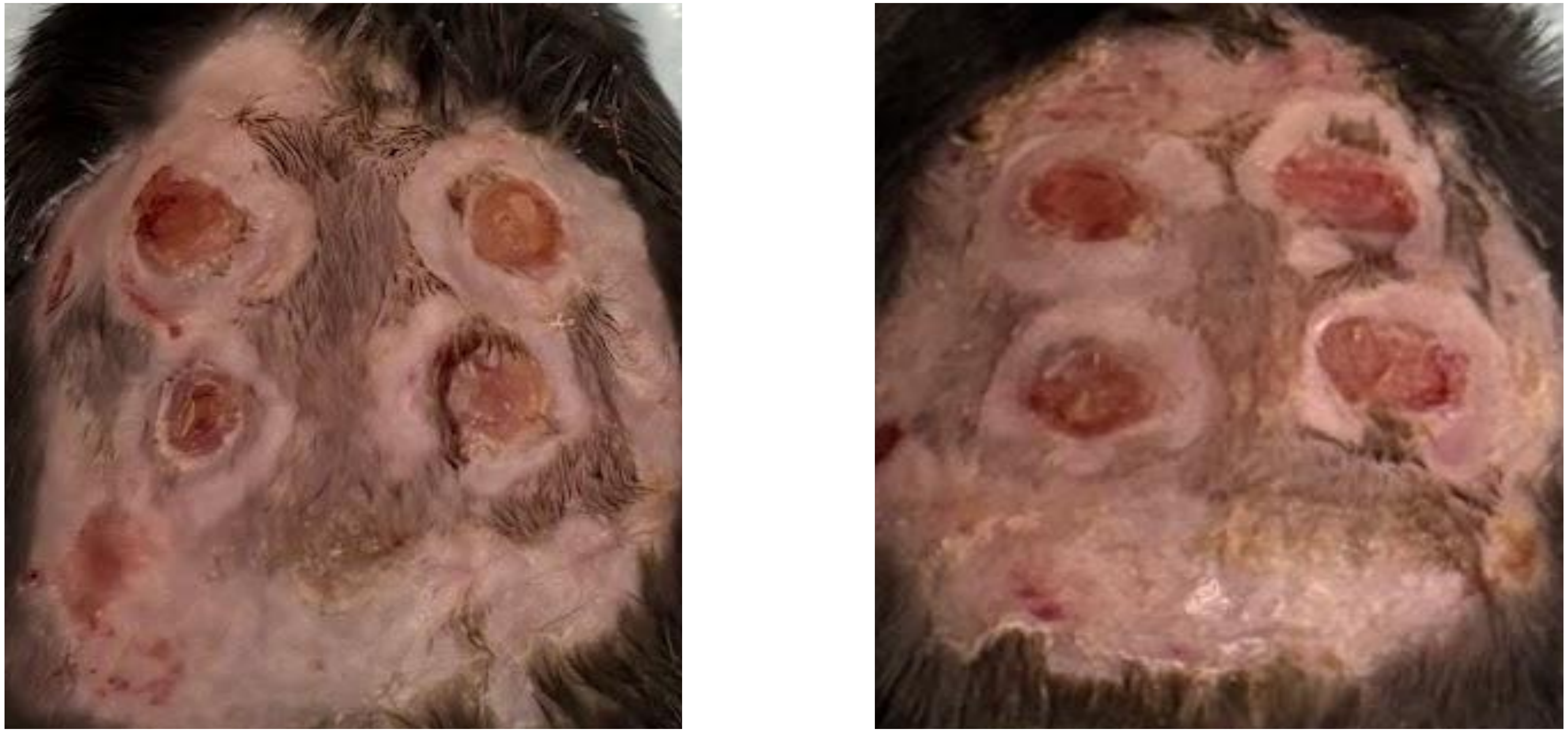

Supplementary Figure S6: Representative images of mouse wounds at time of sacrifice. Left: miR-92a inhibitor-treated mouse, Right: control inhibitor-treated mouse.

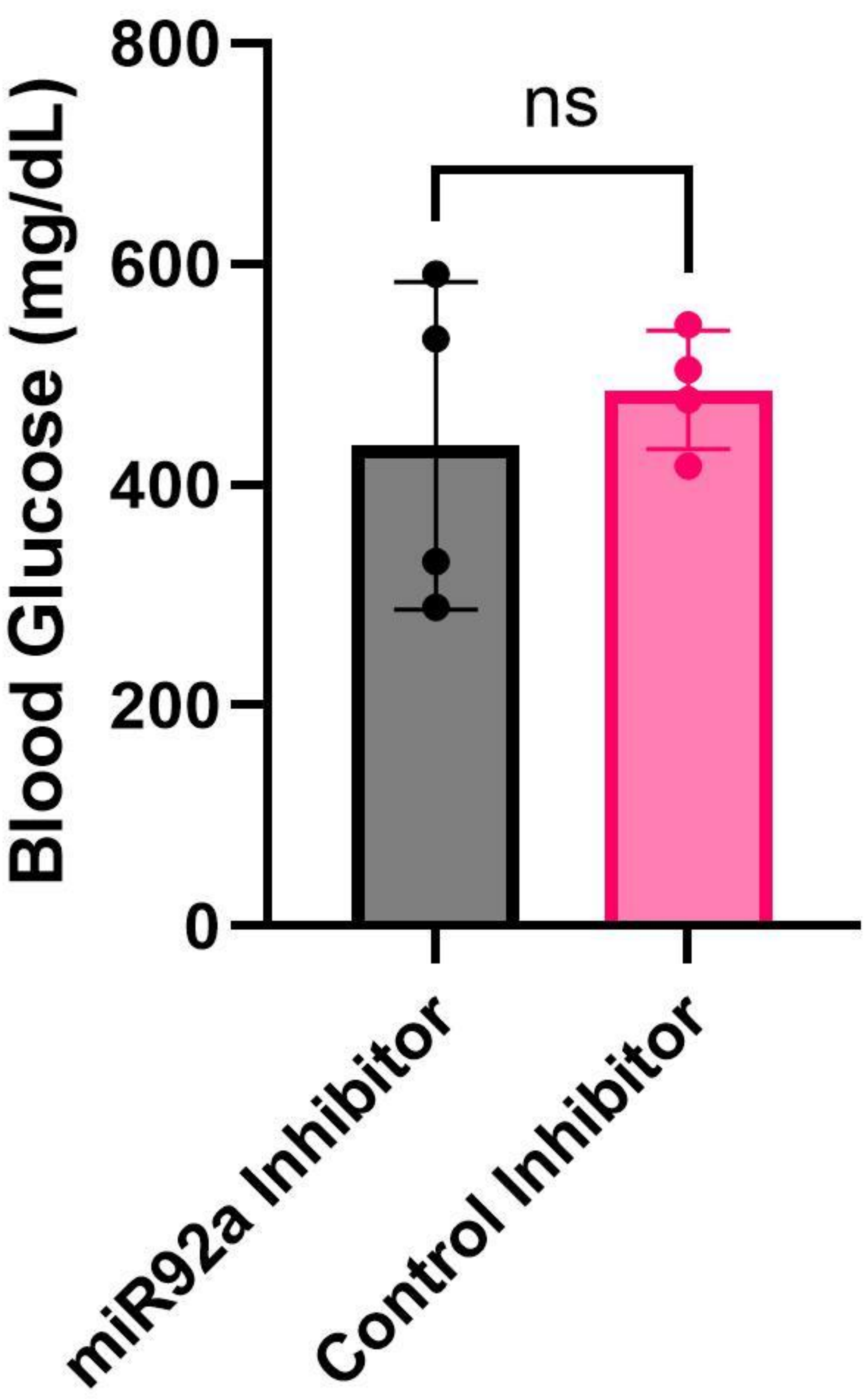

Supplementary Figure S7: Blood glucose of db/db mice used in wound closure experiments measured at sacrifice. Data from mice with blood glucose <250 mg/dL was not used.

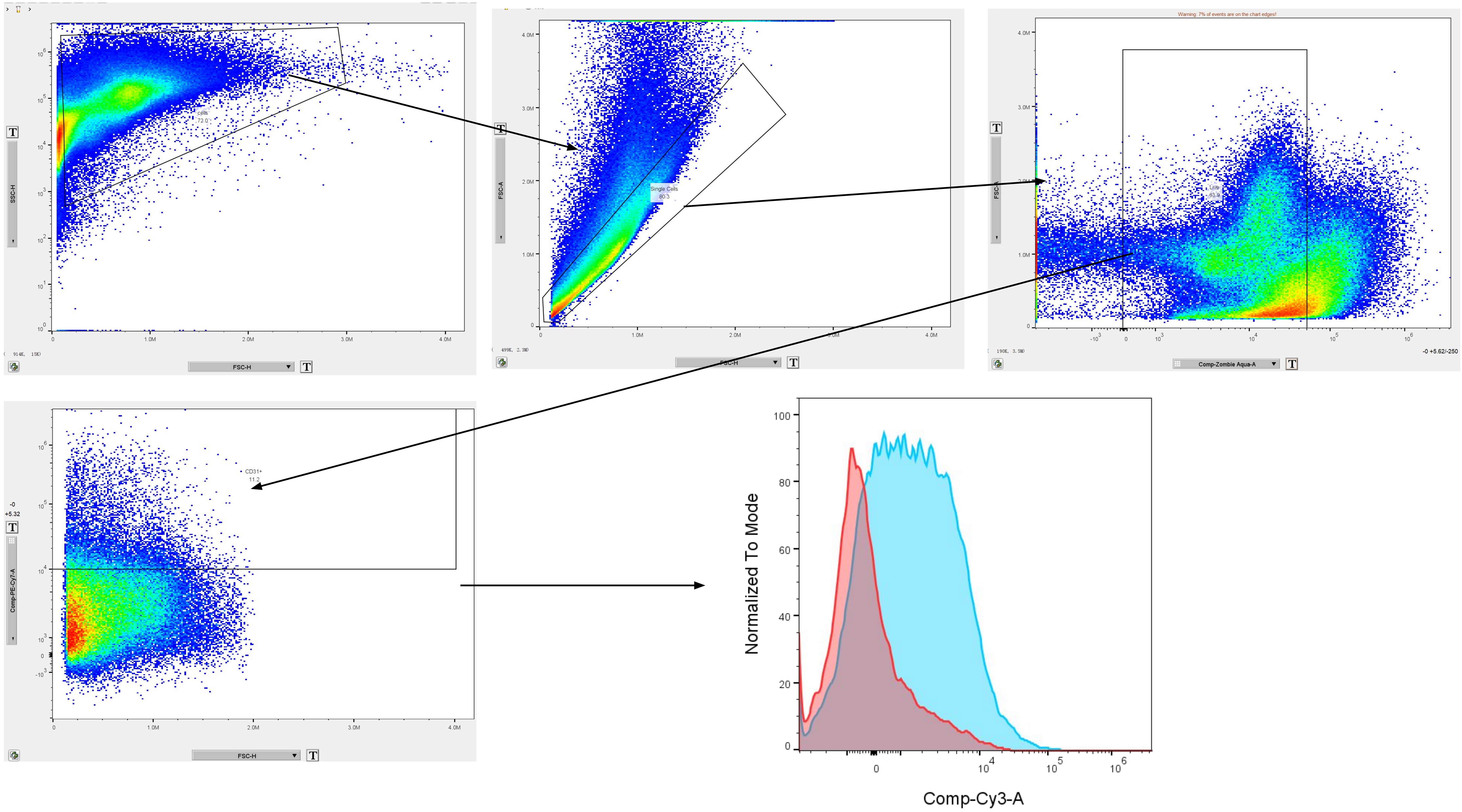

Supplementary Figure S8: Representative gating strategy for CD31 cell uptake

Gene	Forward	Reverse
GAPDH	TGCACCACCAACTGCTTAGC	GGCATGGACTGTGGTCATGAG
UBB	AGTGACGAGAGGCTTTGTCC	CGAAGATCTGCATTTTGACCTGT
KLF2	CCTTCGGTCTTTTCGAGGAC	TAAGGCTTCTCACCTGTGTGTG
ITGA5	GGCACCAGTCCTATCCAGTG	GTGGAGCACATGCCAAGATG

Supplementary Table T1: List of qPCR primers and sequences used.

Antigen	Fluorophore	Dilution	Vendor	Catalog
CD16/32	N/A	1:200	BioLegend	101302
Zombie	Aqua	1:500	BioLegend	423101
CD31	PE-Cy7	1:200	BioLegend	102524
CD45	PerCP-Cy5.5	1:200	BioLegend	157612
CD11b	FITC	1:200	BD	553310
F4/80	BV421	1:100	Biolegend	123131
CD206	BV605	1:100	Biolegend	141721
CD86	BUV737	1:50	BD	741737
EpCAM	PE	1:200	Biolegend	118205
PDGFRa	APC	1:100	Abcam	ab119838
Ly6G	APC-Fire750	1:200	Biolegend	127651

Supplementary Table T2: List of flow cytometry antibodies and reagents used.
